# Draft Genome Sequence of *Escherichia coli* Strain VKPM B-10182, Producing the Enzyme for Synthesis of Cephalosporin Acids

**DOI:** 10.1128/genomeA.01222-14

**Published:** 2014-11-20

**Authors:** Andrey V. Mardanov, Mikhail A. Eldarov, Anna V. Sklyarenko, Maria V. Dumina, Alexey V. Beletsky, Sergey V. Yarotsky, Nikolai V. Ravin

**Affiliations:** a Centre “Bioengineering,” Russian Academy of Sciences, Moscow, Russian Federation; b State Research Institute of Genetics and Selection of Industrial Microorganisms, Moscow, Russian Federation

## Abstract

*Escherichia coli* strain VKPM B-10182, obtained by chemical mutagenesis from *E. coli* strain ATCC 9637, produces cephalosporin acid synthetase employed in the synthesis of β-lactam antibiotics, such as cefazolin. The draft genome sequence of strain VKPM B-10182 revealed 32 indels and 1,780 point mutations that might account for the improvement in antibiotic synthesis that we observed.

## GENOME ANNOUNCEMENT

*Escherichia coli* strain VKPM B-10182 produces a peptidohydrolase specific to the derivatives of phenylacetic acid, tetrazolylacetic acid, and thienylacetic acid. This enzyme, originally named cephalosporin acid synthetase, is able to synthesize antibiotics belonging to β-lactam acids, especially to cephalosporin acids, such as cefazolin. It is very promising for application in the industrial manufacturing of corresponding antibiotics by biocatalytic synthesis (1–3) and in fact is identical to penicillin G acylase (PGA), which is involved in benzylpenicillin hydrolysis (4, 5). Strain VKPM B-10182 is a derivative of strain *E. coli* W (ATCC 9637), which is a well-known PGA producer (4, 5). The mutant strain VKPM B-10182 was produced by multiple rounds of chemical mutagenesis and selection, with the aim of increasing enzyme production and enhancing its specificity toward cephalosporin acid synthesis.

We undertook genome sequencing of this strain in order to identify the genomic variations that formed the molecular basis of the desired phenotype, both for comparative genomic studies and as a prerequisite for constructing its genome-scale metabolic model and facilitating further genetic manipulations for various biotechnological applications.

The draft genome was sequenced using the Roche 454 GS FLX pyrosequencing platform. We obtained a shotgun library of 257,360 single-strand reads, with an average length of 637 bp; the reads were assembled into 74 contigs by the Newbler assembler 2.8 (454 Life Sciences, Branford, CT). The *N*_50_ contig length is 198,256 bp. The draft genome of strain VKPM B-10182 consists of 4.9 Mb, with an average G+C content of 51.6%. For annotation of the draft genome, the Rapid Annotations using Subsystems Technology (RAST) server (6) was applied.

A comparison with the previously sequenced genome of the parental strain ATCC 9637 (7) revealed 32 indels and 1,780 point mutations. Among them, there are 559 synonymous and 988 nonsynonymous substitutions in the protein-coding genes, as well as 233 mutations in noncoding regions. The coding and predicted regulatory regions of the PGA gene in these two strains are identical, indicating that the improvement in enzymatic activity we observed was not due to mutations in the PGA gene but likely resulted from the inactivation of unspecific lactamases or esterases attacking the β-lactam antibiotic nuclei, thus reducing the product yield, or from mutations in some *trans*-acting factors. Plasmid pRK1 (102,536 bp), previously found in strain ATCC 9637 (7), is also present in VKPM B-10182, while the second plasmid, pRK2 (5,360 bp), appeared to be missing in our strain.

A comparative genomics analysis involving strains VKPM B-10182 and ATCC 9637 is under way. Making the genome sequence of *E. coli* VKPM B-10182 available will enable further investigation into the mechanisms of the regulation of PGA biosynthesis and the effect of mutagenesis and selection procedures on the metabolic landscape and gene regulation in the classical model prokaryote.

### Nucleotide sequence accession numbers.

This whole-genome shotgun project has been deposited at DDBJ/EMBL/GenBank under the accession no. JRIA00000000. The version described in this paper is version JRIA01000000.
