# Erratum for Mardanov et al., Draft Genome Sequence of *Escherichia coli* Strain VKPM B-10182, Producing the Enzyme for Synthesis of Cephalosporin Acids

**DOI:** 10.1128/genomeA.00096-15

**Published:** 2015-02-12

**Authors:** Andrey V. Mardanov, Mikhail A. Eldarov, Anna V. Sklyarenko, Maria V. Dumina, Alexey V. Beletsky, Sergey V. Yarotsky, Nikolai V. Ravin

**Affiliations:** a Centre “Bioengineering,” Russian Academy of Sciences, Moscow, Russian Federation; b State Research Institute of Genetics and Selection of Industrial Microorganisms, Moscow, Russian Federation

## ERRATUM

Volume 2, no. 6, e01222-14, 2014. Page 1: In the Acknowledgements: “project ID RFMEFI57614X0061” should read “project ID RFMEFI60414X0022.”

